# Test for the Presence of Mercury

**Published:** 1853-03

**Authors:** 


*Test for the Presence of Mercury.*—If a strong solution of iodide of potassium be added to a minute portion of any of the salts of mercury, placed on a sheet of bright copper, a white metallic silvery stain will be developed, which cannot be mistaken, as no other metal presenting a similar appearance is deposited by the same means. In this way corrosive sublimate may be detected, in a solution unaffected by potash or iodide of potassium. In a mixture of calomel and sugar, in the proportion of one of the former to two hundred of the latter, a distinct metallic stain will be detected with one grain of this mixture, containing but 1-200th of its weight of calomel; and the binoxide of mercury can be thus detected, if mixed with 400 times its weight of sugar. This valuable test we owe to Mr. Arthur Morgan.—*Annals of Pharmacy, from London Journal of Medicine.*

